# Utilizing Light Cure Units: A Concise Narrative Review

**DOI:** 10.3390/polym13101596

**Published:** 2021-05-15

**Authors:** Fatin A. Hasanain, Hani M. Nassar

**Affiliations:** Department of Restorative Dentistry, Faculty of Dentistry, King Abdulaziz University, Jeddah 21589, Saudi Arabia; hnassar@kau.edu.sa

**Keywords:** polymerization, resin composite, light cure, irradiance

## Abstract

The use of photo-curable resin composite restorations is an essential treatment modality in modern dental practice. The success and longevity of these restorations depend on achieving predictable and effective polymerization. Understanding the dynamics of the polymerization and the effect of light cure units (LCUs) on this process is paramount. The goal of this concise narrative review is to provide a simplified presentation of basic principles of composite chemistry, polymerization reactions, and photo-curing with relevant terminologies. Clinical guidelines for choosing and maintaining LCUs, as well as safety precautions and factors under the control of the clinician are listed. Finally, clinical recommendations of LCUs’ usage and monitoring are included to aid practitioners in achieving predictable polymerization during the placement of direct resin composite restorations.

## 1. Introduction

The use of photo-curable resin composite (RC) restorations is increasing, owing to improvements in the mechanical properties, high esthetic outcomes, and the increase in its demand by the population [1,2,3,4]. The steps in the application of such material are relatively standardized. The cavity is prepared based on well-acknowledged conservative principles, followed by the application of the adhesive system, then composite is placed, and then it is light-cured.

The photo-polymerization process is of a great importance because it plays major role in the resultant properties of the final material [5,6]. This is achieved via exposing the uncured resin composite to a source of light which has the energy to initiate the polymerization process. However, the properties of the light are dependent on its source. The efficiency and light quality of the light cure device directly affect the longevity and clinical performance of RC [7]. Many considerations must be taken into account when choosing a light cure unit (LCU). Parameters such irradiance, energy, and beam uniformity are some factors with direct impact on the serviceability of the restorative material. Secondary caries due to the undercuring of RC is among the most commonly cited cause of restoration failures [4,8]. This could be due to less-than-optimal light curing practices leading to inadequate energy delivered and subsequent low degree of conversion (DC) values. It has been reported by many investigators that a lot of practitioners are delivering less energy than required when performing RC restorations [9,10,11,12,13,14]. Unfortunately, many practitioners are unaware of the importance of the light curing process or LCU when they perform a composite restoration [15].

This concise review aims to summarize relevant reports the basic principles of polymerization and photo-curing, as well as the most effective practices of resin composite placement and curing, while elaborating on the types of LCUs currently available and their inherent properties and the characteristics of the accompanied beam. A non-exhaustive literature search was conducted from the year 2000 to 2020. The databases used were Scopus, PubMed, and Google Scholar. The keywords of the search strategy included “dental light curing units”, “irradiance”, “polymerization”, and “dental resin composites”. No specific inclusion or exclusion criteria were applied as to which articles would be included in this review. This narrative review is structured to provide a glimpse of the background information and best practices recommended during resin composite placement, in order to serve as a clinical guide for practitioners and recent dental graduates.

## 2. Resin Composite; Importance and Composition

The applications of RC in dentistry are diverse. This material has been adopted by all dental specialties and has replaced the majority of the metal-based products used for direct restorative work [16,17]. Some practices have shifted to metal-free dentistry, in which all the different purposes are accomplished with tooth-colored materials. Among these, RC can be utilized for the majority of uses such as direct restorations, pits and fissure sealants, temporary restorations, and cements for indirect restorations. The main discussion throughout this review will be focus on the photo-curable RC used for direct restorations. A brief examination of other applications is also included. 

### 2.1. Composition of Resin Composites

The typical light-cured RC formulation contains two distinct components: an organic resin matrix and inorganic filler particles [18]. The resin matrix is the main component, and it plays a major role in the behavior and characteristics of the material. Different variants of monomers are used for the resins with direct restorative purposes. These include bisphenolglycidyl methacrylate (Bis-GMA), urethane dimethacrylate (UDMA), and triethylene glycol dimethacrylate (TEGDMA) comonomer. In addition, novel formulations such as aromatic urethane dimethacrylate (AUDMA) and ethoxylated bisphenol-A dimethacrylate (Bis-EMA) are used in new RC products [19,20,21].

The second major component of RC used in restorative dentistry is the fillers, which includes inorganic oxides and glasses [22,23]. Both the type and the amount of filler suspended in the resin matrix can directly influence the mechanical properties, and consequently the application, of the RC [24]. Among the most common types of filler used are silica and zirconia particles, as well as barium glass and ytterbium trifluorides. These fillers are bonded to the resin matrix constituents with a bi-polar silane coupling agent [25].

In order to initiate the polymerization reaction, a light-sensitive agent, known as a photo-initiator, must be present [26]. These agents are very important in determining the photo-polymerization behavior of the material and will be reviewed later in this review. However, in order to prevent a premature reaction during delivery, storage, and handling during the clinical application, inhibitors such as butylhydroxytoluene (BHT) are used [27]. Additional additives are present in light-cured RC such as pigments and radio-opacifiers, which modulate the shade and the radio-opacity of the material, respectively.

### 2.2. Polymerization of Resin Composites

The first step in the polymerization reaction of light-cured RC material is the creation of active free radical species via light energy supplied by the light cure device [28]. This reactive molecule is termed a photo-initiator compound, and once activated it attacks the double bonds in the resin monomer, converting them into single bonds with electrons available for further reaction of a monomeric unit. The attack of free radicals to the C=C of the monomer is termed the polymerization initiation step, which is followed by the propagation of the polymer chain containing additional monomer units. Each activated monomer unit will be converted, in turn, into a free radical species seeking additional unreacted carbon–carbon double bonds [24,25].

The polymerization reaction continues, and the length of the polymer chain continues to grow, increasing the overall viscosity of the resin matrix medium [5]. This consequently decreases the diffusion of the free radicals and depletes the unreacted monomer, leading to decreasing the rate of polymerization. When two ends of the growing polymer chain react with each other, a termination of the polymerization reaction takes place.

At the end of the polymerization reaction, there was a percentage of C=C remaining which was not converted into single bonds chains within the resultant polymer. The ratio of converted monomer (C–C) to unreacted monomer (C=C) is termed the degree of conversion (DC), and this parameter greatly affects the mechanical and rheological properties of the resultant RC polymer [29,30]. Usually, values for DC for RC materials range between 50% and 75%, with lower DC values associated with low mechanical properties and more chance for material loss due to abrasion, as well as other biological complications, which will be explained in the following sections [31,32,33].

## 3. Dental Light Curing

### 3.1. Light Curing Devices

#### 3.1.1. Quartz–Tungsten Halogen

The first visible LCUs introduced for use in dental clinics were quartz–tungsten halogen (QTH) devices. These units had a bulb which consisted of a tungsten filament encircled in a quartz case. The case was filled with a halogen-based gas. Such units typically required a lot of filtering of the excess heat and visible light, which are not utilized in photocuring [5]. The QTH units are typically hand-held. They also incorporate removable light guides which are hard and non-flexible [34]. These guides allowed a wide range of coverage patterns and improved the ability to reach particular locations within the dental arch [5].

The emission spectrum from QTH units is broad and enables the activation of most types of photo-initiators currently found in dental resin. However, their cooling fans are noisy. The units are also mains powered and deliver a relatively low radiant power and irradiance [35]. Thus, 30–60 s of exposure was required to adequately polymerize a 2 mm increment of dental resin composite [36].

#### 3.1.2. Plasma-Arc Curing

Plasma-arc curing (PAC) LCUs have two tungsten rods at a specific distance from one another [5,36]. The rods are surrounded by an envelope of xenon gas, and emit radiation through a sapphire window. They require a lot of radiation filtering because a large quantity of the radiation falls outside of what is used in dentistry. Their electromagnetic spectrum is wide; thus, they are able to activate all the photo-initiators currently available on the market [5]. Even though PAC LCUs are efficient, they have several disadvantages. They cannot be battery powered and are large, noisy, and expensive. This has led to a decrease in their popularity [37].

#### 3.1.3. Light-Emitting Diodes

Light-emitting diode (LED) LCUs were developed in the late 1990s [36,38]. Compared with QTH units, LEDs have several advantages. LED diodes should last thousands of hours, while QTH bulbs last approximately 30–50 h [39]. LED LCUs also have a higher luminous efficacy in comparison to QTH [40], having light and solid-state emitters. LEDs are currently the most popular type of LCU [40,41,42].

**Figure 1:**
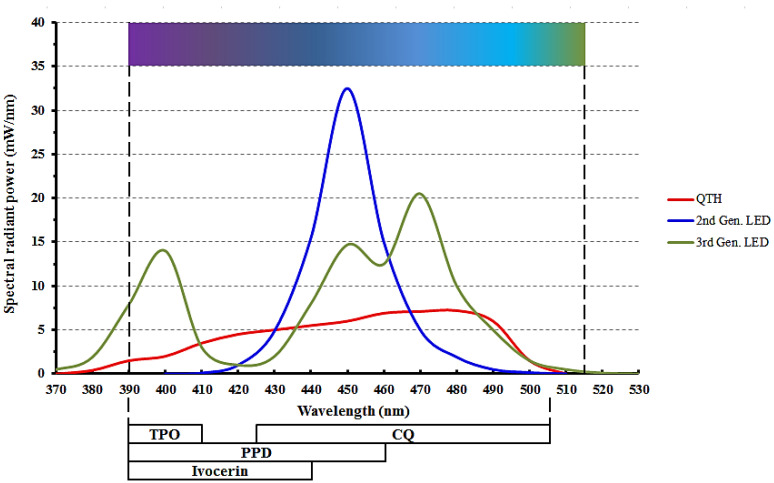
Emission spectra from quartz–tungsten halogen (QTH) and light emitting diode (LED) curing lights. The bars underneath show the relationship of the photo-initiators to the LCU spectra. CQ is Camphorquinone, TPO is diphenyl (2,4,6-trimethylbenzoyl) phosphine oxide, and PPD is phenyl-propanedione. (Adapted from Price, 2017 [37]).

Three generations of LEDs have been developed so far. The first generation of LEDs contained several low-power LEDs. These LCUs had a low output and needed prolonged exposure to cure CQ/TA-based composites in a way which was comparable to QTH available at the time [5,36]. First-generation LED LCUs did not cure dental resin composites as well as QTH could [35,43]. Second-generation LEDs used a single high-power LED which provided a higher light output than the first generation. However, the spectral output was still narrower than that of QTH, similar to that of the first-generation LEDs [35]. Both first- and second-generation LED LCUs are also known as single peak (monowave) LEDs, because they only emit a single color of light (blue) with a wavelength above 420 nm. Lastly, third-generation LEDs have a broader spectral output because of the incorporation of a combination of LEDs. This generation of LCUs is also referred to as multi-wave (multi-peak) LEDs because they emit light of more than one color or wavelength range [37]. Figure 1 shows the spectral profiles of the QTH LCU as well as both second- and third-generation LED LCUs.

### 3.2. Relevant Terms

Most researchers and manufacturers are inconsistent when describing the light output from LCUs [44,45]. Terms such as “power density”, “energy density” and “intensity” are often confusing to researchers and clinicians alike, because their definitions vary from paper to paper [37]. The radiant exposure, LCU output, and wavelength in nm received by the dental resin composite have frequently not been reported [45], which puts the validity of the clinical results from these studies under question [42]. This, in turn, has led to inadvertent spread of incorrect information about LCUs or dental polymer systems which are light-cured [46].

To standardize the description of light from the LCU, it is recommended that the International System of Units (S.I.) be used by manufacturing companies, researchers, and clinicians [42]. Table 1 displays the terms suggested for use by clinicians. The full table can be found in the Halifax symposium [47].

### 3.3. Electromagnetic Spectrum

What appears to humans as light is actually electromagnetic radiation. The spectrum visible to the human eye ranges from the short violet wavelengths (between 390 and 400 nm) to the longer red wavelengths (between 700 and 750 nm) [36]. The infrared spectrum has longer wavelengths than that seen by the human eye, while the ultraviolet range has shorter wavelengths.

For RCs to cure adequately, the photo-initiators must be exposed to the correct wavelength of light. QTH LCUs emit a broad spectrum of both blue and violet light and are capable of activating all photo-initiators used in contemporary dental composites [42]. On the other hand, as mentioned previously, LEDs deliver a narrow range of wavelengths, with many of them not emitting light below 420 nm [44]. When using a single-peak LED, any initiators which need wavelengths below 420 nm are not activated. Multi-peak LEDs do deliver light below 420 nm, which should effectively activate all other commonly used photo-initiators [42].

### 3.4. Photo-Initiators

One of the most common photo-initiator systems used in dental RCs is Camphorquinone/tertiary amine (CQ/TA). Its maximum light absorption occurs at a wavelength of 468 nm and nearly all LCUs can activate it [37,48]. Unfortunately, this photo-initiator system has several disadvantages. CQ is yellow, and once it has been activated, it has an effect on the final color of the restoration, giving it a yellowish tinge. A second disadvantage of using CQ as a photo-initiator is the shorter working time, because the peak absorption in the α-diketone group, derived from CQ, is in the visible light range, causing light from regular light fixtures to initiate the polymerization reaction [36,48].

To resolve the above-mentioned issues related to CQ/TA, companies now incorporate other initiators as well. These include (2,4,6-trimethylbenzoyl) phosphine oxide (TPO) and phenyl-propanedione (PPD) [48]. TPO has been shown to result in a higher degree of conversion as well as improved color stability when compared to a CQ/TA system [49,50]. The color stability makes TPO especially useful when placing extra-white shades of resin composites, often used after tooth bleaching. The absorption spectrum of TPO is from 380 nm to 425 nm, while PPD’s spectrum ranges from below 350 nm to approximately 490 nm [48].

**Figure 2:**
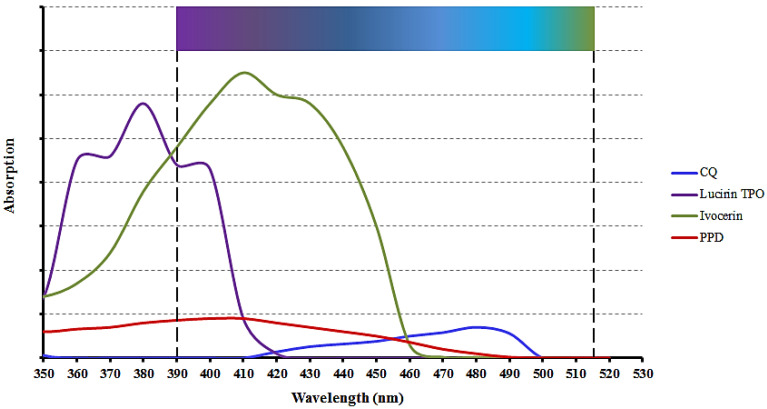
Spectral absorption profiles of common photo-initiators found in resin composite formulations. CQ is Camphorquinone, TPO is diphenyl (2,4,6-trimethylbenzoyl) phosphine oxide, and PPD is phenyl-propanedione. (Adapted from Price, 2017 [37]).

Ivocerin is a new photo-initiator which was developed with the aim of providing a wider spectrum of short-wave absorption. It is a patented germanium derivative and is currently only used in certain Vivadent products [5,51]. The absorption spectrum of Ivocerin is 390–445 nm, with 415 nm being the absorbance maximum [52,53]. The overlap between the absorption profiles of photo-initiators and the spectral profiles of commonly used LEDs is shown in Figure 1. Figure 2 illustrates the absorption profiles of commonly used photo-initiators.

## 4. Factors Affecting Light Cure Irradiance

### 4.1. Radiant Exitance and Irradiance Value

The ISO recommendation for measuring LCU output is by using a laboratory grade power meter. This can be found in the ISO standard 10650:2015. The radiant exitance is then calculated by dividing the total output by the diameter of the tip area [37]. This number is often reported by manufacturers as the irradiance value of the LCU.

When measured at 0 mm away from the LCU tip, it is the same as the term “incident irradiance” used by the SI, as shown in Table 1. This measurement is highly influenced by the distance from the LCU tip, as well as its diameter. The smaller the tip diameter, the larger the radiant exitance value. However, the amount of power remains unchanged. Therefore, in effect, a manufacturer may use a smaller diameter tip to create an LCU which delivers a high irradiance but has a low radiant power output [37]. When a practitioner performs incremental filling, this difference does not have much of an effect on polymerization. However, bulk filling necessitates multiple exposures for adequate polymerization to occur. Thus, it has been recommended that three measurements be reported by manufacturers; active tip diameter, irradiance (mW/cm^2^) and radiant power (Watts) [37].

### 4.2. Active Tip Diameter

The active diameter of the light cure tip is the area from where light is emitted [42]. This area is not the same as the external diameter of the light cure tip and is often unreported by manufacturers or in research papers. If the dental composite being polymerized falls outside the active diameter, then its polymerization is lower [54,55]. Most laboratory studies only evaluate the resin polymerization under the central 4 mm diameter of the LCU tip, not its edges [55]. This further confuses clinicians who may then attempt to cure an entire restoration based on such studies [42]. The clinician may be unaware that to adequately polymerize a large mesio-occluso-distal (MOD) restoration, for example, multiple exposures on the surface of the restoration are required.

### 4.3. Visible Light Curing and Ophthalmological Hazards

All LCUs emit visible light in the blue and blue/violet spectrum. The blue light emitted can cause ocular damage, especially at 440 nm [37,56]. This is true of all dental curing lights. Exposure to high levels of blue light causes irreversible retinal burning immediately as blue light is absorbed by the retina. Even long-term exposure to low levels of blue light accelerates macular degeneration [37,57,58]. Prevention is better than cure. Dental healthcare workers must protect themselves and their patients from the hazards of blue light emitted from LCUs [37,59,60]. It has been found that using high-powered LCUs without the recommended blue light blocker glasses results in the personnel exceeding their maximum recommended exposure to blue light [37,61]. This can happen in as few as seven curing cycles. The use of blue light blocking eye protection prevents both acute and chronic exposure. An appropriate blue light filter, such as the filtering glasses, results in a 99% reduction in the transmission of light with wavelengths less than 500 nm [37]. When orange (amber) glasses are used, a clinician may actually look at the light cure while it is in use, thus ensuring a better light curing procedure during their restorations [13,37,62,63].

### 4.4. Light Beam Uniformity

There is an assumption by clinicians and some researchers that the entire surface at the tip of the LCU emits the same level of light. This assumption was found to be incorrect when the irradiance distribution across the LCU tip was examined using beam profiling methods [37]. The results reported in several studies show that both “cold spots” of low irradiance values and “hot spots” of high values were found across the active tip diameter [44,64,65]. In the clinic, the effect of inappropriately irradiating a part of the final restoration may result in less-than-optimal polymerization, leading to the fracture of stress-bearing areas, such as the marginal ridge [44].

Multi-wave LED LCUs have a lack of homogeneity which lies beyond just hot and cold spots. Different areas across the tip may have very different emissions of violet (400–410 nm) or blue light (450–470 nm) [66,67]. The effect of all this inhomogeneity can negatively affect the microhardness and degree of conversion of the final restoration [67,68]. These negative effects may be partially overcome by increasing the total exposure time beyond the manufacturers’ recommended time [44]. It is also suggested that the clinician be knowledgeable of the beam profile of the LCU in use. If the information is not available, a lack of homogeneity should be assumed and the area should be covered more than once with the active tip to ensure adequate polymerization.

### 4.5. Effect of Light Cure Tip to Resin Distance

Simply put, the irradiance received decreases as the LCU is moved further away from the restorative material [40,69]. However, the effect varies because light delivery is not the same with different LCUs. The effect is not even homogenous within the same LCU due to lack of beam uniformity; some light is dispersed, while some is more collimated [37]. Some researchers have suggested that an increase in exposure time from 20 to 60 s will allow sufficient exposure for a deep proximal box, even in high-intensity LCUs [39,69]. Clinicians need to be aware that increasing exposure time does cause heat generation in the dental tissues [70,71].

Manufacturers report several output characteristics. Table 2 shows the output characteristics as reported by their manufacturers. Ideally, they should report both the irradiance exitance as well as the irradiance delivered at up to 10 mm away [42]. This is not the case for all manufacturers; therefore, the clinician should increase exposure time as the LCU moves further away from the restoration being cured.

### 4.6. Effect of Infection Control Barrier

LCUs are a potential source of infection in the dental clinic because the same LCU is used on several patients. Thus, an infection control barrier is vital for the prevention of cross-contamination. The clinician must be aware, however, that the use of an infection control barrier may reduce the delivered irradiance from the LCU [72]. One study found that light curing sleeves or even plastic wrap did not significantly affect irradiance. However, the use of gloves or other opaque barriers had a significant effect [73]. Another study found that there was a reduction of 5–8% when using disposable barriers such as sleeves and plastic food wrap [74]. This number increased if the barriers were used incorrectly, such as the seam covering the active tip. Thus, the use of clear barriers is recommended, and it wise to measure the irradiance with the barrier in place to ensure that the total output is still of a clinically acceptable level [37,42].

**Figure 3:**
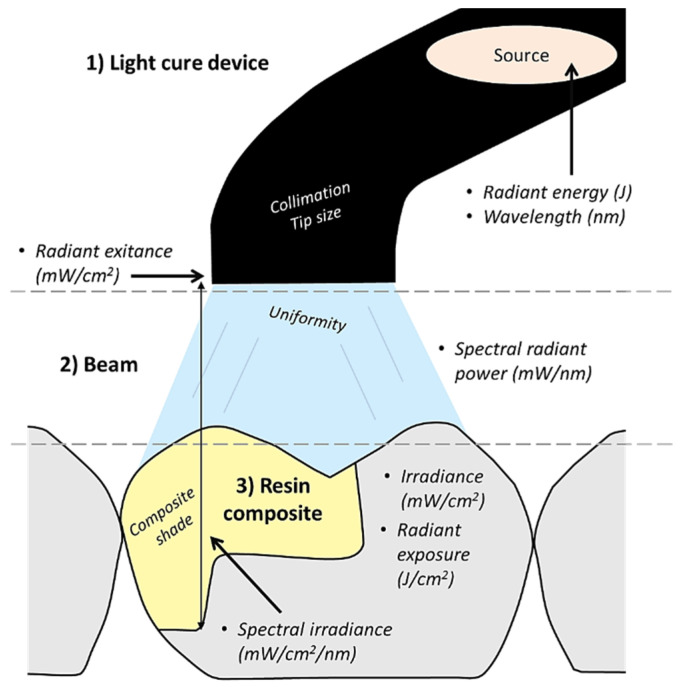
A diagrammatic illustration of the process of light curing a resin composite restoration including the relevant terminologies and influencing parameters.

There are four sets of variables which determine success in placing RCs. These are curing light characteristics, operator technique, restoration characteristics, and the energy requirements of the dental resin composite used. These variables created the acronym CORE, and the term CORE checklist is used to determine success when placing RCs [75,76]. Figure 3 diagrammatically displays the variables discussed so far.

## 5. Sequelae for Improper Light Curing

As explained previously, DC is a very important parameter that governs the properties of the final RC material [29,30]. Typically, high DC values are needed to ensure that the majority of the monomer is converted into polymer chains. The cross-linkage of the resulting polymer is reflected as high mechanical properties (e.g., compressive strength and fracture toughness). Consequently, low values for DC are indicative of low polymeric cross-linking and low mechanical properties. The DC is highly affected by the number of free radicals readily available during the propagation phase of the polymerization reaction, which in turn is affected by the amount of energy is subjected to the monomer [30,32]. This is highly correlated to the performance of the light-cure device which should provide a light beam with specific characteristics in order to ensure predictable conversion of the monomer into a polymer, thereby maximizing the DC.

**Figure 4:**
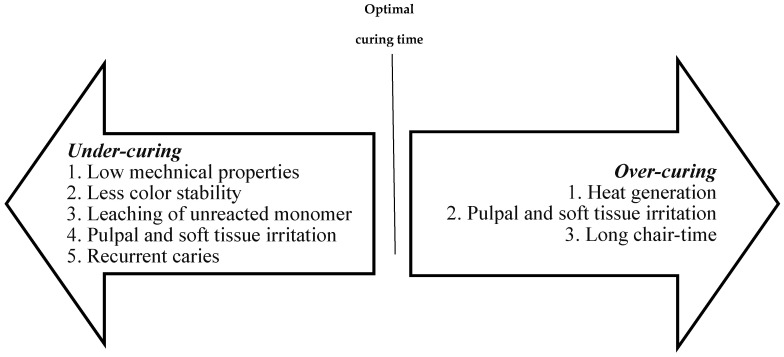
A diagram listing the common sequale of under- and over-curing of resin composites.

In addition to low mechanical properties and subsequent low abrasion resistance of the material, low DC values at deeper layers of the restoration and at the tooth-level can lead to microleakage and subsequent recurrent caries development [4,8]. Recurrent caries can necessitate the replacement of composite restorations, which accounts for 60% of procedures performed in dental offices [77]. Furthermore, low DC indicates more unreacted monomer in the final restoration, which could leach out into dentinal tubules causing pulpal inflammation in deep cavities and soft tissue irritation in cavities close to gingival tissues [78,79]. Figure 4 illustrates the effects of under- and over-curing RC.

There is high risk of superficial tissue damage with light cure devices with >1200 mW/cm^2^ irradiance values [71]. Additionally, the authors conclude that the risk of pulpal damage is more likely with these irradiance values, especially in deep cavities and exposure times between 20 and 30 s.

There are some modifications of the conventional RC formulations which have been introduced to satisfy some particular dental applications. Flowable composites and resin-based fissure sealants are used as a preventive measure against the development of dental caries in occlusal surfaces of posterior teeth with deep and retentive grooves [80]. The former can also be used as a liner in some situations. These materials tend to have low filler content compared to the resin matrix content. This allows better penetration of curing light throughout the material. In addition, these materials are usually placed in thin layers and are considered less prone to complications of inadequate light curing devices. However, they tend to have lower mechanical properties owing to the reduced filler content [81].

In contrast, the new bulk-fill composite materials are highly susceptible to inadequate light curing because they require high amount of energy in order to deliver sufficient energy to guarantee adequate DC at deeper levels of the material. The primary reason for introducing bulk-fill formations is to shorten the chair-time by enabling the bulk filling of cavities up to 5 mm in a single increment without the need for the incremental placement of RC [82,83,84]. However, in order to achieve this, the light curing beam should be able to deliver adequate energy through the bulk of the material. Consequently, LCDs with irradiance values greater than 1000 mW/cm^2^ are recommended by manufacturers [31,82]. Table 3 shows several different types of bulk-fill composite materials currently available on the market.

A third specific application is the use of resin-modified glass ionomer liners as indirect pulp-capping materials [30]. These materials are typically placed in deeper cavities, approximating the pulp, in order to protect the pulp from chemical irritation of the unreacted monomer of RC as well as to isolate the pulp from heat generation by the LCD during the polymerization process [85,86]. Although these materials are placed in thin layers, the fact that they are placed in cavities with deep pulpal floors far away from the LCD tip could potentially lead to less energy delivery and subsequent non-optimal DC.

## 6. Best Light Curing Practices

**Figure 5:**
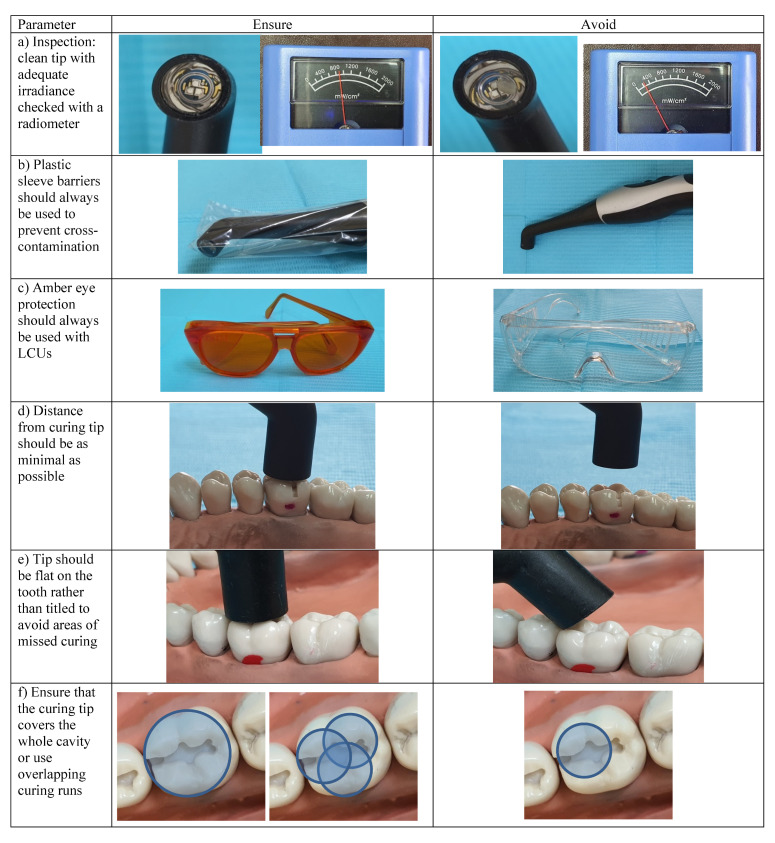
Best practices used during light curing resin composite restorations: (**a**) inspection of LCU tip cleanliness and output; (**b**) the use of disposable plastic barriers to avoid cross-contamination between patients; (**c**) amber protective eye wear or shield should always be used; (**d**) the light cure device tip must be as close as possible to the surface of the restoration; (**e**) the tip must be perpendicular to the restoration to be cured in order to avoid areas missing exposure; (**f**) light cure device tip must be large enough to cover the entire area of the restoration. Otherwise, use overlapping runs.

**Figure 6:**
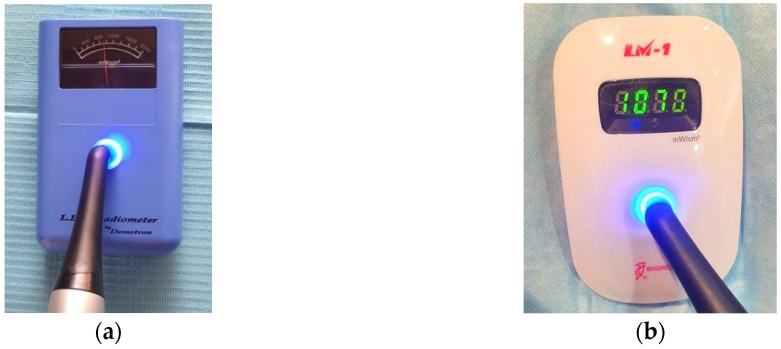

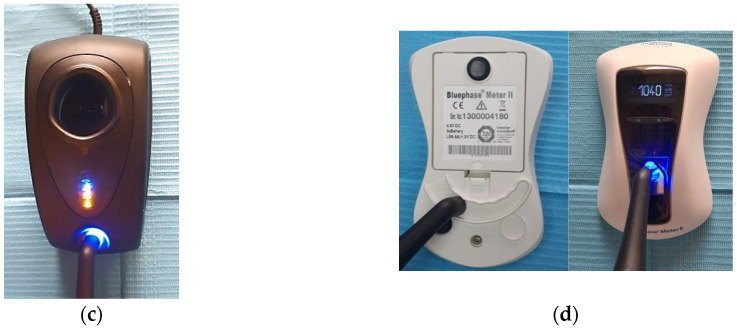
Types of dental radiometers: (**a**) analogue; (**b**) digital; (**c**) built into the light cure device’s bases, and (**d**) digital with tip size gauge that influence the irradiance reading.

Curing RC restoration is a vital step for a successful, long lasting restoration. In the dental clinic, several practical tips which help ensure the curing process is as efficient and effective as possible are presented in Table 4 and Figure 5. Maintaining the LCU with frequent check-ups using radiometers is vital in ensuring both successful and long-serving restorations. Figure 6 shows some of the commonly used dental radiometers available.

## 7. Conclusions

A vital part of a dental practitioner’s arsenal in the dental operatory is an awareness and understanding of the LCU used, along with its limitations. These include, but are not limited to, the type of LCU unit used and how well it will polymerize a particular dental resin composite material based on the wavelength(s) of LCU and the photo-initiators in the material, the amount of radiant energy emitted by the LCU at a given point in time and its effect on the final restoration, as well as the effect of distance from the LCU on the final polymerization process, and whether that distance is due to cavity depth or operator handling. LCUs also require regular maintenance, without which the result of clinical procedure is likely to be compromised.

LCUs play a critical role in the longevity of a restoration, all else being equal. By being aware of the LCU used in the dental operatory along with its specific limitations, a clinician may ensure a better match between the LCU and restorative material placed. Using the clinical tips presented in the best practices section will also help to ensure that the restoration placed is as well polymerized as possible.

## Figures and Tables

**Table 1:** Glossary of relevant terminology used for light curing (adapted from Platt and Price, 2014 [47]).

Term	Unit Commonly Used in Dentistry	Symbol	Notes/Significance of Term
Radiant energy	Joule	J	This describes the energy from the curing light source
Radiant exposure (fluence)	Joule per cubic centimeter	J/cm^3^	It describes the energy emitted or received
Radiant exitance (or radiant emittance)	Milliwatt per square centimeter	mW/cm^2^	Radiant power/flux emitted from a defined area. To be used instead of power density or irradiance when describing the output from a curing light and is influenced by tip diameter
Irradiance (incident radiation)	Milliwatt per square centimeter	mW/cm^2^	Radiant power on the surface
Spectral radiant power	Milliwatt per nanometer	mW/nm	Radiant power emitted per wavelength of light. Longer wavelengths have less energy than shorter wavelengths. Higher power usually needs shorter exposure time, while lower power requires longer exposure time
Spectral irradiance	Milliwatt per square centimeter per nanometer	mW/cm^2^/nm	Irradiance received by the resin at each nm. The further away the LCU tip, the less irradiance received

**Table 2:** Manufacturers’ reported output settings of commonly used dental curing lights.

Light Cure Device	Manufacturer Details	Wavelengths (nm)	Curing Tip Diameter (mm)	Modes	Irradiance (mW/cm^2^)	Built-In Radiometer
Elipar DeepCure-S	3M ESPE, St. Paul, Minnesota, USA	430–480 monowave	10	Standard	1470	No
Bluephase PowerCure	Ivoclar Vivadent, Schaan, Liechtenstein	385–515 multiwave	9	PreCure Turbo High power 3 s	950 1200 2000 3000	Yes
VALO Cordless	Ultradent Products, South Jordon, Utah, USA	385–515 multiwave	Not disclosed	Normal High power Xtra power	1000 1400 3200	No
Demi Ultra	KaVo Kerr, Orange, California, USA	450–470 monowave	8	Standard	1100–1330	Yes
SmartLite Pro	Dentsply Sirona, Konstanz, Germany	Cure tip: 450–480 PolyCure tip: 405–480 monowave	10	Standard	1200	Yes

**Table 3:** Information about different bulk-fill resin composite materials used for direct restorations.

Material	Main Monomer	Main Fillers	Photo-Initiator	Manufacturer
Filtek Bulk-Fill	AUDMA	Silane-treated ceramics	CQ	3M ESPE, Dental Products, Saint Paul, MN, USA
Tetric Evo-Ceram Bulk Fill	Bis-EMA	Barium aluminium silicate glass	CQ, Ivocerin^®^	Ivoclar Vivadent, Zurich, Switzerland
Tetric N-Ceram Bulk Fill	Bis-GMA	Barium aluminium silicate glass	CQ, Ivocerin^®^	Ivoclar Vivadent, Zurich, Switzerland
SonicFill	3-trimethoxysilylpropyl methacrylate	Barium glass	CQ	Kerr Dental, Orange, CA, USA
Beautiful Bulk Restorative	Bis-GMA	S-PRG fluoroboroaluminosilicate glass	Not disclosed	Shofu Inc., Kyoto, Japan
X-tra fil	MMA	Inorganic fillers	Not disclosed	Voco, Cuxhaven, Germany
SureFil SDR *	UDMA	Barium glass	CQ	Dentsply Caulk, Milford, DE, USA
Filtek Bulk Flow *	Bis-GMA	Silane treated ceramic, ytterbium fluoride filler	CQ	3M ESPE, Dental Products, Saint Paul, MN, USA
Tetric Evo-Flow Bulk Fill *	Dimethacrylates	Barium glass	CQ, Ivocerin^®^	Ivoclar Vivadent, Zurich, Switzerland
Venus Bulk-Fill *	UDMA	Barium glass	Not disclosed	Heraeus Kulzer, South Bend, IN, USA
Beautifil Bulk Flowable *	Bis-GMA	S-PRG fluoroboroaluminosilicate glass	Not disclosed	Shofu Inc., Kyoto, Japan
EverX Posterior *	Bis-GMA	Barium borosilicate glass	CQ	GC Dental Products, Tokyo, Japan
X-tra base *	Bis-EMA	Inorganic fillers	Not disclosed	Voco, Cuxhaven, Germany
MI Fil *	UDMA	Silica nanofillers	Not disclosed	GC Dental Products, Tokyo, Japan

* Flowable composite.

**Table 4:** Recommended practices for optimal LCU utilization during composite placement.

Recommended Practice	Reference(s)
Choose the LCU which matches the photo-initiators in the RC material. LEDs are the most commonly used LCUs; therefore, the use of multi-wave LEDs is preferred because it activates all currently used photo-initiators.	Price, 2010 [76] Price, 2014 [87] Rueggeberg et al., 2017 [5] Price, 2017 [37]
Prior to each LCU use, check the tip for cleanliness. Any debris on the tip affects the light curing process and should be removed prior to curing the restoration.	Ajaj et al., 2018 [72] Suliman et al., 2019 [88]
Ensure that the infection control barrier is used and placed correctly, with no seam covering the active tip diameter.	Rueggeberg et al., 2017 [5] Price, 2017 [37] Ajaj et al., 2018 [72]
The use of light-blocking glasses is strongly advocated because they nearly eliminate the blue light hazard.	Rueggeberg et al., 2017 [5] Shortall et al., 2016 [15] Price, 2017 [37]
During the use of the LCU inside the patient’s mouth, position the LCU as close as possible to the restoration surface and place it as flat as possible to gain optimal curing. Compromised access and darker shades of composite should be compensated for by increasing the curing time.	Shortall et al., 2016 [15] Price, 2017 [37].
Ensure that the active curing tip covers the entire restoration. If it is smaller than the restoration, several overlapping runs will be needed to attain adequate polymerization of the RC.	Shortall et al., 2016 [15] Price, 2017 [37] Price et al., 2020 [42].
The use of a dental radiometer to monitor the LCU in practice is a quick and easy way to ensure that the LCU is still emitting the required irradiance Regular monitoring also allows the practitioner to know when the irradiance has dropped and LCU unit needs maintenance or replacement.	Rueggeberg et al., 2017 [5] Assaf et al., 2020 [89] Price et al., 2012 [90].
